# Characteristics of salivary microbiota in children with obstructive sleep apnea: A prospective study with polysomnography

**DOI:** 10.3389/fcimb.2022.945284

**Published:** 2022-08-29

**Authors:** Xin Huang, Xuehui Chen, Xu Gong, Ying Xu, Zhifei Xu, Xuemei Gao

**Affiliations:** ^1^ Department of Orthodontics, Peking University School and Hospital of Stomatology, Beijing, China; ^2^ Department of Respiratory Medicine, Beijing Children’s Hospital, Capital Medical University, National Center for Children’s Health, Beijing, China

**Keywords:** Pediatric obstructive sleep apnea, salivary microbiota, 16S rRNA gene sequencing, polysomnography, adenotonsillectomy

## Abstract

**Objectives:**

The present study aimed to investigate the characteristics of salivary microbiota of children with obstructive sleep apnea (OSA) and to assess longitudinal alterations in salivary microbiota before and after adenotonsillectomy.

**Methods:**

A set of cross-sectional samples consisted of 36 OSA children (17 boys and 19 girls, 7.47 ± 2.24 years old) and 22 controls (9 boys and 13 girls, 7.55 ± 2.48 years old) were included in the study, among which eight OSA children (five boys and three girls, 8.8 ± 2.0 years old) who underwent treatment of adenotonsillectomy were followed up after 1 year. Saliva samples were collected, and microbial profiles were analyzed by bioinformatics analysis based on 16S rRNA sequencing.

**Results:**

In cross-sectional samples, the OSA group had higher α-diversity as estimated by Chao1, Shannon, Simpson, Pielou_e, and observed species as compared with the control group (*p* < 0.05). β-Diversity based on the Bray–Curtis dissimilarities (*p* = 0.004) and Jaccard distances (*p* = 0.001) revealed a significant separation between the OSA group and control group. Nested cross-validated random forest classifier identified the 10 most important genera (*Lactobacillus*, *Escherichia*, *Bifidobacterium*, *Capnocytophaga*, *Bacteroidetes_[G-7]*, *Parvimonas*, *Bacteroides*, *Klebsiella*, *Lautropia*, and *Prevotella*) that could differentiate OSA children from controls with an area under the curve (AUC) of 0.94. Linear discriminant analysis effect size (LEfSe) analysis revealed a significantly higher abundance of genera such as *Prevotella* (*p* = 0.027), *Actinomyces* (*p* = 0.015), *Bifidobacterium* (*p* < 0.001), *Escherichia* (*p* < 0.001), and *Lactobacillus* (*p* < 0.001) in the OSA group, among which *Prevotella* was further corroborated in longitudinal samples. *Prevotella sp_HMT_396* was found to be significantly enriched in the OSA group (*p* = 0.02) with significantly higher levels as OSA severity increased (*p* = 0.014), and it had a lower abundance in the post-treatment group (*p* = 0.003) with a decline in each OSA child 1 year after adenotonsillectomy.

**Conclusions:**

A significantly higher microbial diversity and a significant difference in microbial composition and abundance were identified in salivary microbiota of OSA children compared with controls. Meanwhile, some characteristic genera (*Prevotella*, *Actinomyces*, *Lactobacillus*, *Escherichia*, and *Bifidobacterium*) were found in OSA children, among which the relationship between *Prevotella* spp. and OSA is worth further studies.

## Introduction

Pediatric obstructive sleep apnea (OSA) refers to a breathing disorder characterized by recurrent, partial, or complete episodes of upper airway obstruction and can result in neurobehavioral and cardiovascular complications and growth impairment, commonly associated with intermittent hypoxemia and sleep fragmentation (Marcus et al., 2012; Savini et al., 2019).

The main cause of pediatric OSA is adenotonsillar hypertrophy (Arens and Marcus, 2004). Johnston and Douglas suggested that a large number of pathogenic bacteria existed in hypertrophic tonsils and adenoids (Johnston and Douglas, 2018). Previous studies have explored the pathogenic bacteria of adenoids or tonsils in diseases such as tonsillitis (Johnston et al., 2018), otitis media (Nistico et al., 2011; Dirain et al., 2017), and rhinosinusitis (Stapleton et al., 2021), while these studies paid more attention to the microbiota of adenoids and tonsils with recurrent or chronic infection.

However, alterations in microbiota have been proposed to be related to OSA (Cai et al., 2021). Evidence from animal models showed that gut microbiota could be affected by intermittent hypoxia (Moreno-Indias et al., 2015; Tripathi et al., 2019), and alterations of the microbiota could also elicit sleep disturbances (Badran et al., 2020), which might further mediate OSA-related morbidities (Durgan et al., 2016; Xue et al., 2017). Meanwhile, human studies indicated the potential role of the microbiome in the pathophysiology of OSA (Wu et al., 2019; Yang et al., 2019) and OSA-associated morbidities (Ko et al., 2019a; Ko et al., 2019b; Chen et al., 2022). To date, there have been relatively few studies on the microbiota of OSA children, some of which were concerning gut microbiota (Collado et al., 2019; Valentini et al., 2020) and others concerning adenotonsillar microbiota (Galli et al., 2020; Kim and Min, 2020), while studies on oral microbiota in OSA children were few (Xu et al., 2018).

Oral microbiota, the second most abundant microbiota after the gastrointestinal tract, is an important part of human microbiota (Verma et al., 2018). It communicates with the nasopharynx and oropharynx, constitutes an entry point to the digestive system, and was a source of microbial down-transmission (Schmidt et al., 2019; Wang et al., 2020; Willis and Gabaldón, 2020). Additionally, studies have shown that children with mouth-breathing had a drier mouth, a lower oral pH value, a higher level of free sialic acid, and poorer oral health when compared to healthy children (Weiler et al., 2006; Grillo et al., 2019; Tamasas et al., 2019; Davidovich et al., 2021). Thus, the disturbed oral environment of OSA children might have corresponding microbial characteristics.

In the present study, we hypothesized that there were significant differences in salivary microbiota between OSA children and healthy controls. We aimed to investigate the characteristics of salivary microbiota in OSA children by 16S rRNA gene sequencing and validate it by longitudinal alterations 1 year after adenotonsillectomy.

## Materials and methods

### Study design

**Figure 1:**
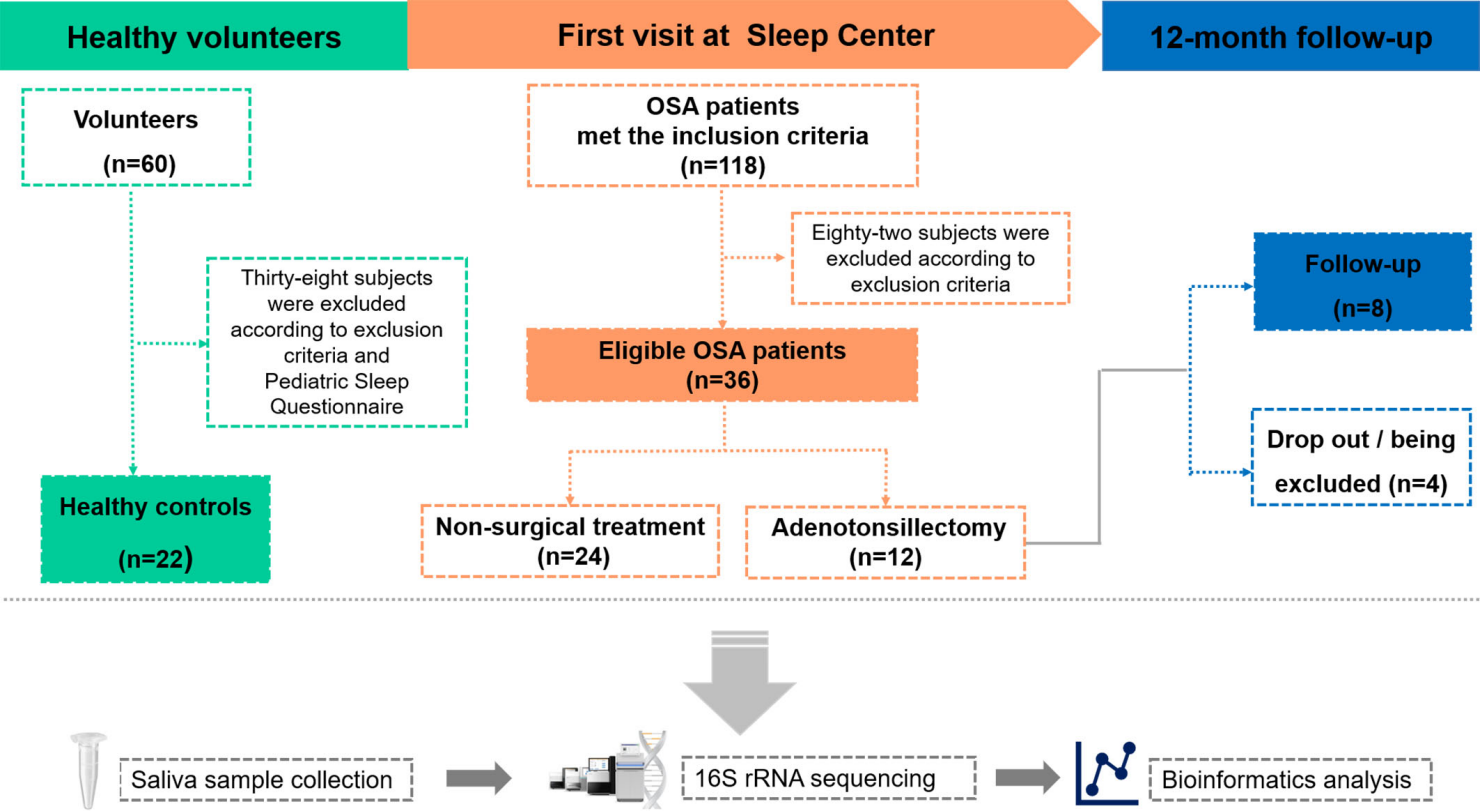
Overview of the study design and sample collection. PSG, polysomnography; OSA, obstructive sleep apnea.

This study was made up of two parts. The first part was a cross-sectional study, which compared the salivary microbiota differences between OSA children and controls. The second part was a longitudinal study, which compared the salivary microbiota differences in OSA children before and after OSA treatment. The study design is shown in **Figure 1**.

### Study population

Micropower package (Kelly et al., 2015), a simulation-based method for permutational multivariate analysis of variance (PERMANOVA)-based β-diversity comparisons, was applied to assess the effect size and statistical power of this study. The Human Microbiome Project (HMP) dataset was used for distance matrix simulation (Creasy et al., 2021). The effect sizes (ω^2^) were calculated using the simulated matrixes of 80% and 90% powers for varying sample numbers per group (**Supplementary Table 1**). Compared with the effect size in published microbiome studies cataloged by Kelly et al. (Kelly et al., 2015), the effect size in a sample size of 20 subjects per group with an ω^2^ of 0.027 is smaller than the effects observed in studies of antibiotic exposure that were analyzed by unweighted the Jaccard distances. Thus, 20 subjects per group likely afford adequate statistical power for the primary outcome measure.

The OSA samples were from pediatric patients who were referred to the Department of Sleep Center of Beijing Children’s Hospital for standard in-lab polysomnography (PSG) by otolaryngologists from January to June 2020. The inclusion criteria for the OSA group were as follows: 1) age between 6 and 12 years, 2) pediatric OSA confirmed by overnight PSG, 3) diagnosed with adenoid hypertrophy and (or) tonsillar hypertrophy by otolaryngologists, 4) mouth-breathing reported by a parent or legal guardian, and 5) informed consent to participate in the study.

The control group comprised family members of hospital staff in Peking University School and Hospital of Stomatology, aged between 6 and 12 years. In the corresponding period, they were screened by the Mandarin version of the Pediatric Sleep Questionnaire–Sleep-Related Breathing Disorder (PSQ-SRBD) completed by a parent or legal guardian.

For each subject, current and past medical history was collected, and oral examinations were performed by two qualified dentists (one examined while the other recorded) in our team. The exclusion criteria for both the OSA group and control group were as follows: 1) intake of antibiotics and anti-infammatory drugs in the last 3 months; 2) other known factors associated with pediatric OSA including craniofacial anomalies, overweight, and obesity according to body mass index (BMI) cutoffs for Chinese children and adolescents (Li et al., 2010) and neuromuscular disease; 3) systemic diseases, autoimmune diseases, and congenital disorders; 4) history of infections in the last 3 months; 5) history of tonsillectomy and (or) adenoidectomy; 6) decayed tooth, periodontitis, oral mucosal diseases, ongoing dental treatments, and usage of mouthwashes regularly; 7) with a particular diet; and 8) the presence of pets in the home. For the longitudinal study, the exclusion criteria for follow-up children were the same as above (with the exception of criteria for a history of tonsillectomy and adenoidectomy).

A total of 58 children were included in the cross-sectional study (**Table 1**). There were 36 children in the OSA group, including 19 girls and 17 boys, aged 7.47 ± 2.24 years. In the control group, there were 22 children, 13 girls and 9 boys, aged 7.55 ± 2.48 years. A total of eight children were followed up 1 year after treatment and were included in the longitudinal study (**Table 2**). PSG was re-examined, and saliva samples were collected again during the follow-up.

**Table 1:** Demographic and polysomnography data of children in OSA group and control group.

	OSA group (n = 36)	Control group (n = 22)	*p*-Value
Age, years†	7.47 ± 2.24	7.55 ± 2.48	0.908
Sex, male§	17 (47.2%)	9 (40.9%)	0.639
BMI, kg/m^2^‡	17.4 (15.0, 22.1)	16.5 (14.5, 18.1)	0.106
AHI, events/h	3.8 (2.6, 6.9)	n.d.	–
OAI, events/h	0 (0, 0.3)	n.d.	–
OAHI, events/h	2.7 (1.4, 5.3)	n.d.	–
MTAD, s	12.2 (10.8, 14.5) (min–max: 0–17.1)	n.d.	–
MOAD, s	0 (0, 12.7) (min–max: 0–20.4)	n.d.	–
MHD, s	19 (15.6, 22.3) (min–max: 12.8–31.1)	n.d.	–
ODI, events/h	2.2 (0.9, 3.2)	n.d.	–
Lowest SaO_2_, %	91 (88, 94)	n.d.	–

BMI, body mass index; OSA, obstructive sleep apnea; AHI, apnea–hypopnea index; OAHI, obstructive apnea–hypopnea index; MTAD, mean total apnea duration; MOAD, mean obstructive apnea duration; MHD, mean hypopnea duration; ODI, oxygen desaturation index; SaO_2_, oxygen saturation; n.d., not determined.

† Mean ± standard deviation with Student’s t-test.

‡ Median (first quartile, third quartile) with Mann–Whitney U-test.

§ Frequency (percentage) with chi-square test.

**Table 2:** Demographic and polysomnography data of OSA children before and 1 year after adenotonsillectomy.

	Pre-treatment group(n = 8)	Post-treatment group(n = 8)	*p*-Value
Age, years†	8.8 ± 2.0	9.8 ± 2.0	0.33
Sex, male§	5 (62.5%)	5 (62.5%)	1.00
BMI, kg/m^2^‡	19.2 (14.7–22.6)	18.6 (14.7–24.3)	0.674
AHI, events/h‡	5.8 (3.6–14.3)	1.6 (0.7–2.7)	0.012*
OAI, events/h‡	0.5 (0.1–1.3)	0 (0–0)	0.018*
OAHI, events/h‡	3.5 (1.6–8.7)	0.6 (0.3–1.9)	0.017*
MTAD, s‡	13.1 (12.2, 14.8) (min–max: 9.8–15.7)	13.1 (11.5, 14.3) (min–max: 9.5–16.4)	0.721
MOAD, s‡	13.1 (9.2, 14.2) (min–max: 0–20.4)	0 (0, 0) (min–max: 0–0)	0.002**
MHD, s‡	17.2 (15.5, 18.8) (min–max: 12.8–21.7)	13.5 (10.8, 14.9) (min–max: 0–16)	0.007**
ODI, events/h‡	2.7 (2.2–9.3)	1.0 (0.3–1.4)	0.012*
Lowest SaO_2_, %‡	91.5 (85.0–92.0)	93.0 (90.5–94.0)	0.011*

Note. BMI, body mass index; OSA, obstructive sleep apnea; AHI, apnea–hypopnea index; OAHI, obstructive apnea–hypopnea index; MTAD, mean total apnea duration; MOAD, mean obstructive apnea duration; MHD, mean hypopnea duration; ODI, oxygen desaturation index; SaO_2_, oxygen saturation.

† Mean ± standard deviation with Student’s t-test.

‡ Median (first quartile, third quartile) with Mann–Whitney U-test.

§ Frequency (percentage) with chi-square test.

* p < 0.05, ** p < 0.01.

### Examinations of obstructive sleep apnea

All the children in the OSA group were examined for overnight PSG. Prior to PSG, they were examined for adenoids and tonsils by otolaryngologists in the outpatient clinic, which indicated that adenotonsillar hypertrophy was the main cause of OSA in our participants on the basis that we have excluded children with overweight, obesity, craniofacial abnormalities, and neuromuscular disease. OSA children followed up were re-examined for PSG 1 year after adenotonsillectomy.

The overnight PSG examinations were performed using a Compumedics E-series PSG System (Compumedics, Abbotsford, VIC, Australia) or Alice 5 Diagnostic Sleep System (Respironics, San Diego, CA, USA). PSG was interpreted by two technicians and one pediatrician trained in sleep medicine who were unaware of the clinical findings. Respiratory events were scored as obstructive apnea or hypopnea according to the criteria of the American Academy of Sleep Medicine (Berry et al., 2012). The obstructive apnea–hypopnea index (OAHI) was calculated as the average number of obstructive apneas and hypopneas per hour of sleep, and the diagnosis of OSA was confirmed by OAHI ≥ 1 events/h (Sateia, 2014). Mild OSA is diagnosed with 1 < OAHI ≤ 5 events/h, moderate OSA with 5 < OAHI ≤ 10 events/h, and severe OSA with OAHI > 10 events/h (Kaditis et al., 2017).

### Screening of controls with pediatric sleep questionnaire–sleep-related breathing disorder

PSQ-SRBD is a currently available diagnostic instrument and epidemiological tool (Chervin et al., 2000). It has been introduced and localized into the Mandarin version of PSQ-SRBD and has been used and validated in a large epidemiological survey in mainland China (Li et al., 2016; Li et al., 2018). It was suggested that the PSQ-SRBD was suitable for use in Chinese children with acceptable sensitivity and specificity in screening children with probable OSA when the cutoff score is 7 points (Li et al., 2016; Li et al., 2018). In the present study, 60 healthy volunteers were recruited. Twenty-two children with scores less than 3 were regarded as children with the lowest risk of sleep-disordered breathing and were included in the control group.

### Saliva sample collection

A saliva sample collection procedure was conducted according to the microbiome sampling protocol of the Human Microbiome Project (McInnes and Cutting, 2010). All saliva samples were collected following the same protocol. Saliva samples of the OSA group were collected at 6:00–7:00 a.m. right after finishing PSG monitoring at the Sleep Center of Beijing Children’s Hospital. Participants were asked to sit in a comfortable position and not to brush their teeth, drink, or eat to minimize the potential risk of contamination. The participants were then instructed to let saliva collect in the mouth for 1 min and then drool into the labeled 50-ml collection tube (Falcon, sterile conical polypropylene tube with flat-top screw cap). This process may be repeated multiple times in order to collect 1.5 ml of unstimulated saliva. Saliva samples of the control group were collected following the same protocol as mentioned above at the participants’ own homes. After collection, the samples were immediately placed on ice and transferred to the laboratory within 1 h. All the samples were equalized to the volumes of 1 ml by pipettes and then were centrifuged at 10,000 ×*g* for 10 min at 4°C. The pellets were stored at −80°C before DNA extraction.

### DNA extraction and 16S rRNA gene sequencing

Total genomic DNA extraction was performed using QIAamp DNA mini kit (Qiagen, Hilden, Germany) according to the manufacturer’s instructions. Detailed protocols were as follows: 1) suspend bacterial pellet in 180 μl of lysozyme (20 mg/ml) followed by incubation at 37°C for 30 min to lyse the rigid multilayered cell wall (Wade and Prosdocimi, 2020). 2) Add 20 μl of proteinase K solution (600 mAU/ml) followed by incubation at 56°C for 30 min. 3) Add 40 μl of RNase A (10 mg/ml) followed by incubation at room temperature for 10 min. 4) Add 200 μl of Buffer AL to the sample followed by incubation at 70°C for 10 min. 5) Supplement the sample with 200 μl of ethanol (96%) before being loaded onto the QIAamp Mini spin column and centrifuge at 8,000 ×*g* for 1 min. 6) Wash the column with 500 μl of buffer AW1 and 500 μl of buffer AW2. 7) DNA was eluted with 40 μl of buffer AE. The quantity and quality of extracted DNAs were measured using a NanoDrop ND-1000 spectrophotometer (Thermo Fisher Scientifc, Waltham, MA, USA) and agarose gel electrophoresis, respectively. The V3–V4 regions of the 16S rRNA gene were amplified and sequenced on an Illumina NovaSeq instrument using paired-end 2 × 250 bp sequencing at Shanghai Personal Biotechnology Co., Ltd (Shanghai, China). Samples were sequenced in the same run to prevent batch effects.

### Bioinformatics and statistical analysis

As for bioinformatics analysis, the sequence data were analyzed using QIIME2 (Bolyen et al., 2019) and R packages (v3.2.0). Raw sequences were processed with the Divisive Amplicon Denoising Algorithm 2 (DADA2) plugin to generate amplicon sequence variants (ASVs) (Callahan et al., 2016). Compared with traditional operational taxonomic units (OTU) clustering-based methods, DADA2 used a model-based, clustering-free approach to correct amplicon errors and group unique sequences into ASVs. Therefore, it is thought to identify taxonomic variations at a finer scale. Taxonomy was assigned to ASVs against the Human Oral Microbiome Database (I et al., 2020).

α-Diversity indexes (Chao1, Shannon, Simpson, Pielou_e, and observed species) were calculated based on the ASV table and visualized as box plots. β-Diversity analysis was performed using the Bray–Curtis dissimilarities (abundance weighted distance) and Jaccard distances (the presence/absence of detected ASVs—not abundance weighted) and visualized *via* principal coordinate analysis (PCoA) and unweighted pair group method with arithmetic mean (UPGMA) clustering tree. The signifcance of differentiation of microbiota structure between groups was assessed by PERMANOVA and Permdisp (Permutational analyses of multivariate dispersions). Linear discriminant analysis (LDA) effect size (LEfSe) analysis was performed to detect differentially abundant taxa between groups and visualized as cladogram and LDA score histogram. LDA was used to assess the effect size of each feature. The cutoff value of the LDA score (log10) was 2. The nested cross-validated random forest classifier was built by QIIME2. Receiver operating characteristic (ROC) analysis was conducted based on the top 10 important genera. The performance of the model was measured as area under the curve (AUC).

Statistical analysis was performed with SPSS (version 23.0; IBM). The Mann–Whitney U-test was performed to determine signifcant differences with respect to continuous variables. A chi-square test was used for the assessment of the association of frequencies between groups. Unless otherwise stated, *p* < 0.05 was regarded as indicating statistical significance.

## Result

### Description of participants

A total of 36 OSA children and 22 healthy controls were enrolled in the study (**Table 1**). In the OSA group, median apnea–hypopnea index (AHI) and OAHI were 3.8 events/h and 2.7 events/h, respectively. The median for mean total apnea duration (MTAD) and mean hypopnea duration (MHD) were 12.2 s and 19 s, respectively. The median oxygen desaturation index (ODI) and lowest oxygen saturation were 2.2 events/h and 91%, respectively. There was no significant difference in demographic characteristics between the OSA group and control group.

Eight OSA children who underwent adenotonsillectomy were followed up after 1 year of treatment (**Table 2**). One year after adenotonsillectomy, the median OAHI decreased from 3.5 to 0.6 events/h. The median for MOAD decreased from 13.1 to 0 s, and the median for MHD decreased from 17.2 to 13.5 s. The median lowest oxygen saturation increased from 91.5% to 93%.

### Comparisons of salivary microbiota between pediatric obstructive sleep apnea patients and controls

**Figure 2:**
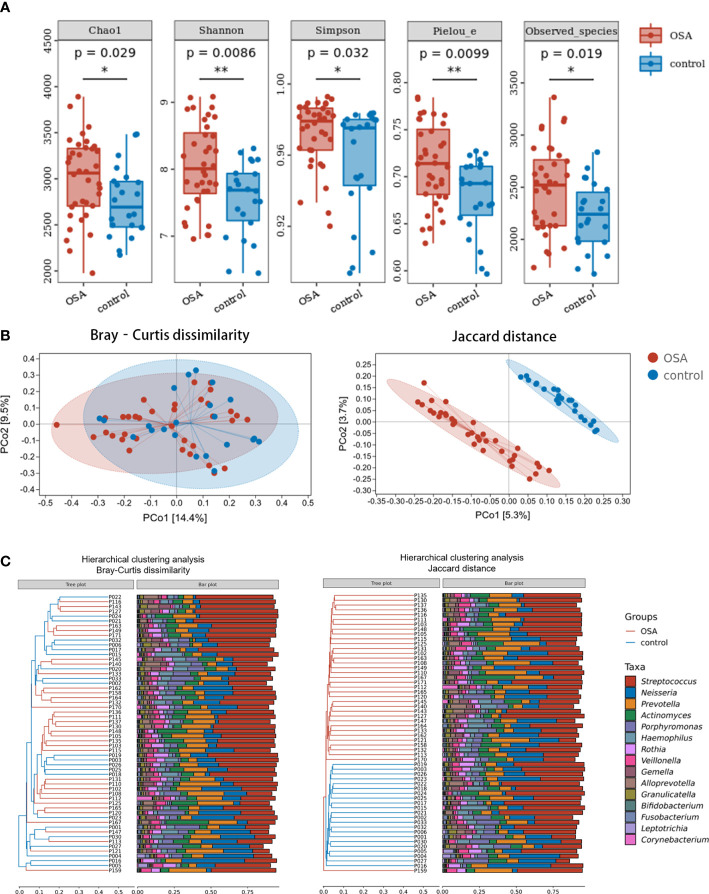
Diversity and composition of salivary microbiota in pediatric obstructive sleep apnea (OSA) patients and controls. **(A)** Box plots showing α-diversity estimators in OSA group and control group (*p* < 0.05, Mann–Whitney U-test). **(B)** PCoA (principal coordinate analysis) plot of bacterial β-diversity based on Bray–Curtis dissimilarity (abundance‐based) and Jaccard distance (composition‐based). Numbers between square brackets represent the percentage of the total variance explained by principal coordinates. **(C)** The UPGMA (unweighted pair group method with arithmetic mean) clustering tree based on Bray–Curtis dissimilarity and Jaccard distance at genus level. The branch length represents the distance between the samples. If the community composition of the samples is similar, they are clustered in a cluster tree. * *p* < 0.05, ** *p* < 0.01.

Compared with healthy controls, OSA patients had significantly higher α-diversity as estimated by Chao1, Shannon, Simpson, Pielou_e, and observed species (**Figure 2A**, *p* < 0.05, Mann–Whitney U-test). β-Diversity between samples was visualized by PCoA and UPGMA clustering tree (**Figures 2B, C**). PCoA revealed that salivary microbiota of OSA patients and controls had a significant separation based on the Bray–Curtis dissimilarities (*p* = 0.004, PERMANOVA) and Jaccard distances (*p* = 0.001, PERMANOVA). Permutational analyses of multivariate dispersions (PERMDISP) was also performed to further test the homogeneity of within-group multivariate dispersions. The result indicated that the intra-group variances were not significantly different based on the Bray–Curtis dissimilarities (*p* = 0.707, PERMDISP) and Jaccard distances (*p* = 0.373, PERMDISP).

When analyzing β-diversity based on the Bray–Curtis dissimilarities (abundance-weighted distances), we found that the separation in the PCoA plot was not clear, although there was a statistical difference between the two groups (**Figure 2B**). Similarly, samples from the OSA group and control group were mixed in the UPGMA clustering tree (**Figure 2C**). However, when analyzing β-diversity based on the Jaccard distances (composition-weighted distances), there was a significant separation in the PCoA plot as well as a statistical difference (**Figure 2B**). Similarly, samples from the OSA group and control group were clustered correctly in the UPGMA clustering tree except for one sample (**Figure 2C**). This result indicated that the difference in salivary microbiota between OSA children and controls was more significant when the bacterial composition was considered alone.

### Identification of characteristic salivary microbiota in pediatric obstructive sleep apnea

**Figure 3:**
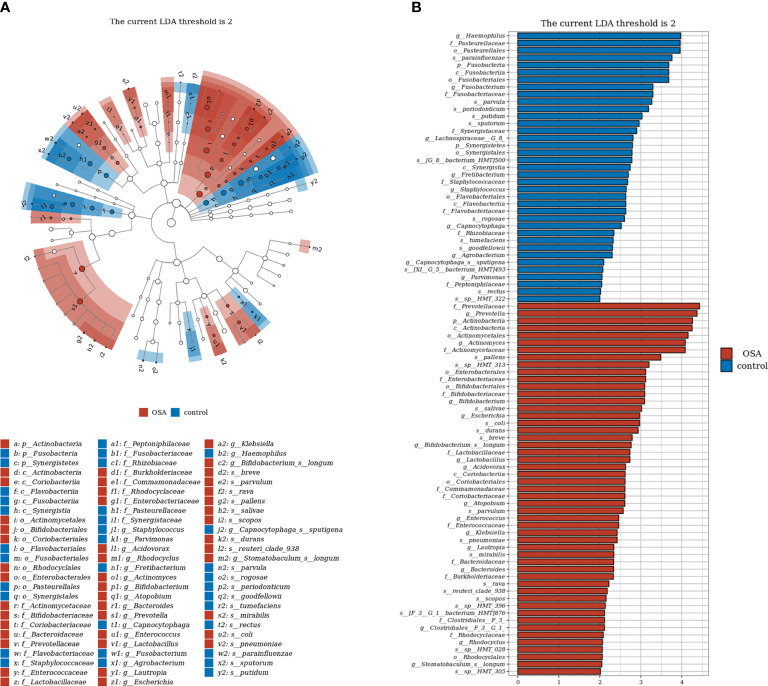
Linear discrimination analysis (LDA) effect size (LEfSe) analysis on the differential taxa between obstructive sleep apnea (OSA) group and control group. **(A)** In the LEfSe taxonomic cladogram, the colored nodes from inner circle to outer circle represent the hierarchical relationship of all taxa from the phylum to species level. Taxa enriched in OSA group are shown in red, and taxa enriched in control group are shown in blue. Taxa with non-significant changes are colored in white. The diameter of each small circle represents the taxa abundance. Only the LDA scores > 2 are listed. **(B)** Histogram showing 88 taxa with significant difference detected between OSA group and control group. Of these taxa, 51 taxa were enriched in OSA group, and 37 taxa were enriched in control group. The greater the LDA score was, the more significant the microbiota was in the comparison. Only the LDA scores > 2 are listed.

**Figure 4:**
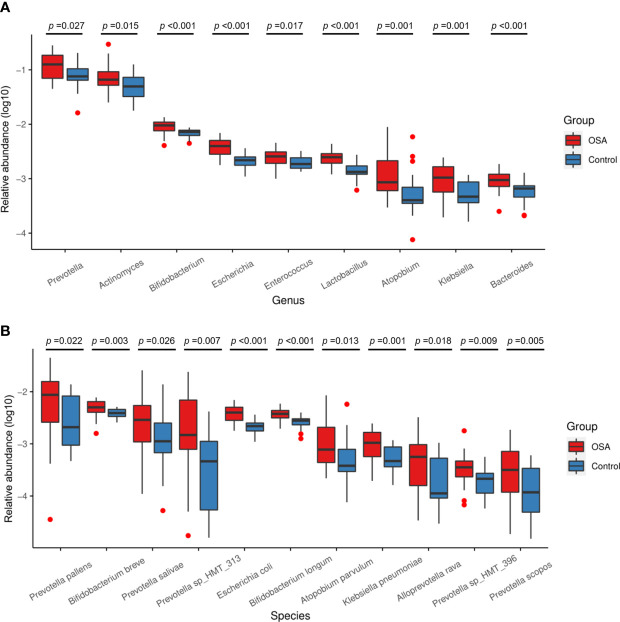
Relative abundance of nine genera and 11 species enriched in obstructive sleep apnea (OSA) group verified by linear discriminant analysis effect size (LEfSe) analysis. Box plots showing log10-transformed relative abundances of **(A)** genera and **(B)** species with significant enrichments in OSA group. Boxes represent the interquartile ranges. Lines inside the boxes denote medians. Taxa are ranked in order of relative abundance from highest to lowest. *p*-Values from Mann–Whitney U-test are indicated in the figure.

**Figure 5:**
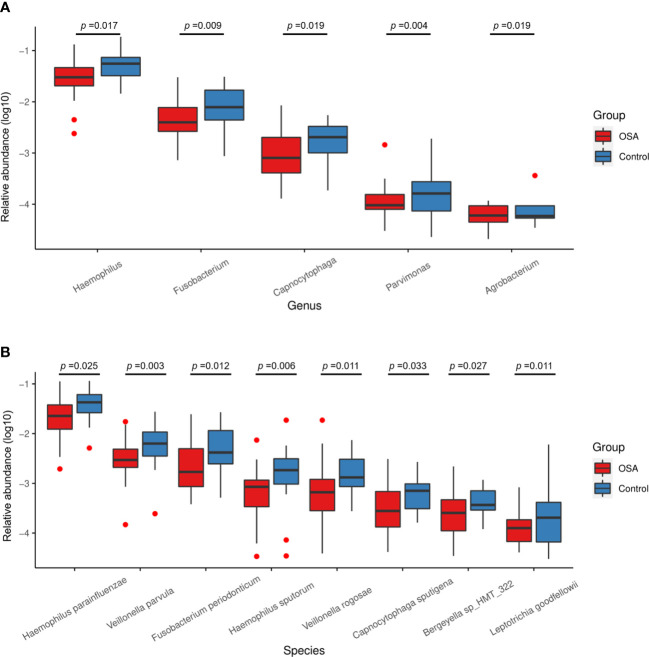
Relative abundance of five genera and eight species enriched in control group verified by linear discriminant analysis effect size (LEfSe) analysis. Box plots showing log10-transformed relative abundances of **(A)** genera and **(B)** species with significant enrichments in control group. Boxes represent the interquartile ranges. Lines inside the boxes denote medians. Taxa are ranked in order of relative abundance from highest to lowest. *p*-Values from Mann–Whitney U-test are indicated in the figure.

To further investigate the salivary microbial features of pediatric OSA patients, we performed LEfSe analysis to select taxa with significant differences between the OSA group and control group using the criteria of LDA scores > 2 and *p* < 0.05 (**Figure 3**). As a result, 88 taxa with a significant difference were detected between the OSA group and control group. Of these taxa, 51 taxa were enriched in the OSA group, and 37 taxa were enriched in the control group. The relative abundance of 9 genera and 11 species, which were significantly enriched in the OSA group (**Figure 4**), and that of 5 genera and 8 species, which were significantly enriched in the control group (**Figure 5**), were summarized and shown as box plots.

Salivary microbiota of OSA patients was mainly characterized by a significantly higher abundance of genera such as *Prevotella* (*p* = 0.027), *Actinomyces* (*p* = 0.015), *Bifidobacterium* (*p* < 0.001), *Escherichia* (*p* < 0.001), *Enterococcus* (*p* = 0.017), *Lactobacillus* (*p* < 0.001), *Atopobium* (*p* = 0.001), *Klebsiella* (*p* = 0.001), and *Bacteroides* (*p* < 0.001) as compared with controls (**Figure 4A**). In terms of differences in species between groups, we found that many species in the genus *Prevotella* such as *Prevotella pallens* (*p* = 0.022), *Prevotella salivae* (*p* = 0.026), *Prevotella sp_HMT_313* (*p* = 0.007), *Prevotella sp_HMT_396* (*p* = 0.009), and *Prevotella scopos* (*p* = 0.005) were significantly enriched in the OSA group (**Figure 4B**). Furthermore, other species such as *Bifidobacterium breve* (*p* = 0.003), *Escherichia coli* (*p* < 0.001), *Bifidobacterium longum* (*p* < 0.001), *Atopobium parvulum* (*p* = 0.013), *Klebsiella pneumoniae* (*p* = 0.001) were also found to have a significantly higher abundance in the OSA group (**Figure 4B**).

In healthy children, genera such as *Haemophilus* (*p* = 0.017) and *Fusobacterium* (*p* = 0.009) had higher abundance (**Figure 5A**). In terms of species (**Figure 5B**), healthy children were characterized by a higher abundance of *Haemophilus parainfluenzae* (*p* = 0.025), *Veillonella parvula* (*p* = 0.003), *Fusobacterium periodonticum* (*p* = 0.012), *Haemophilus sputorum* (*p* = 0.006), and *Veillonella rogosae* (*p* = 0.011).

**Figure 6:**
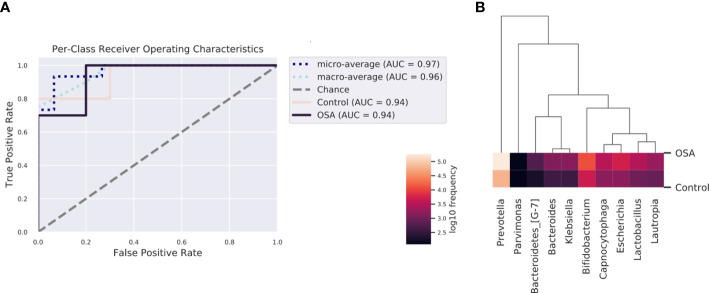
Receiver operating characteristic (ROC) analysis of combined salivary bacterial biomarkers. **(A)** ROC curve of top 10 genera identified by nested cross-validated random forest classifier. Micro-averaging calculates metrics globally by averaging across each sample. Macro-averaging gives equal weight to the classification of each sample. **(B)** Heatmap of top 10 important genera identified by nested cross-validated random forest classifier.

To determine whether the salivary microbiota can be regarded as identification biomarkers for distinguishing OSA patients from healthy children, we built a nested cross-validated random forest classifier at the genus level and identified the 10 most important genera that differentiate the OSA and control groups. These genera, in descending order of importance, were *Lactobacillus* (feature importance = 0.25), *Escherichia* (feature importance = 0.16), *Bifidobacterium* (feature importance = 0.11), *Capnocytophaga* (feature importance = 0.09), *Bacteroidetes_[G-7]* (feature importance = 0.08), *Parvimonas* (feature importance = 0.08), *Bacteroides* (feature importance = 0.07), *Klebsiella* (feature importance = 0.06), *Lautropia* (feature importance = 0.05), and *Prevotella* (feature importance = 0.05). ROC curve analysis was performed, and the results showed that OSA patients could be accurately distinguished from healthy controls as indicated by the AUC, which had a value of up to 0.94 (**Figure 6**).

### Effects of adenotonsillectomy on salivary microbiota

**Figure 7:**
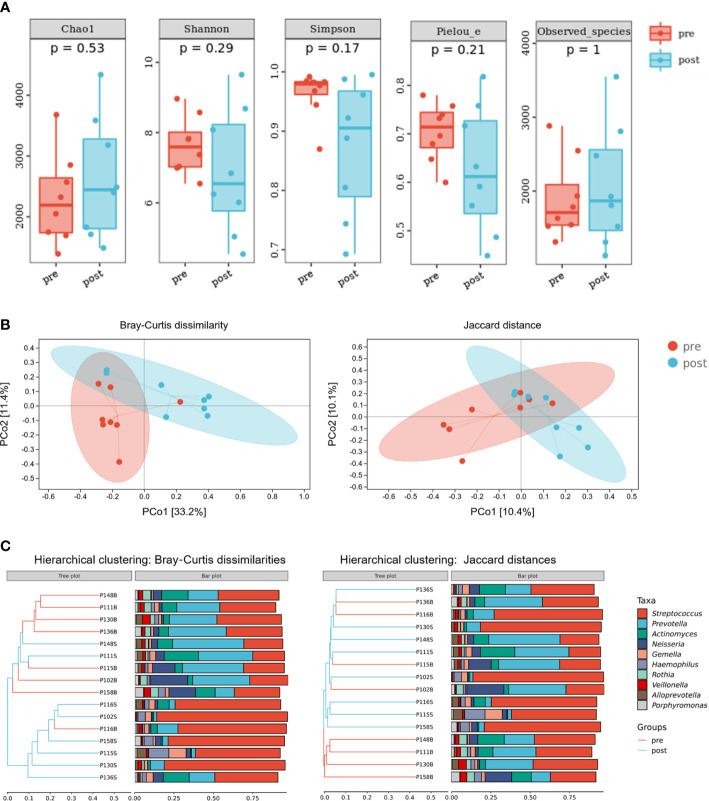
Diversity and composition of salivary microbiota in pediatric obstructive sleep apnea (OSA) patients before and 1 year after adenotonsillectomy. **(A)** Box plots showing α-diversity estimators measured for eight OSA patients pre- and post-treatment (*p* > 0.05, Mann–Whitney U-test). **(B)** PCoA (principal coordinate analysis) plot of bacterial β-diversity based on Bray–Curtis dissimilarity (abundance‐based) and Jaccard distance (composition‐based). Numbers between square brackets represent the percentage of the total variance explained by principal coordinates. **(C)** The UPGMA (unweighted pair group method with arithmetic mean) clustering tree based on Bray–Curtis dissimilarity and Jaccard distance at genus level. The branch length represents the distance between the samples. If the community composition of the samples is similar, they are clustered in a cluster tree.

To evaluate the effects of adenotonsillectomy on salivary microbiota, we conducted a 1-year follow-up of OSA children who underwent adenotonsillectomy. Saliva sampling was repeated following the same protocol, and samples from the pre-treatment group and post-treatment group were sequenced in the same run to prevent the batch effect. The results showed that there were no significant differences in α-diversity as estimated by Chao1, Shannon, Simpson, Pielou_e, and observed species (**Figure 7A**, Mann–Whitney U-test, *p* > 0.05). PCoA revealed that salivary microbiota of OSA patients and controls had a significant separation based on the Bray–Curtis dissimilarities (*p* = 0.017, PERMANOVA) and Jaccard distances (*p* = 0.064, PERMANOVA), indicating that it was the abundance, not the composition, that changed significantly 1 year after adenotonsillectomy (**Figure 7B**).

**Figure 8:**
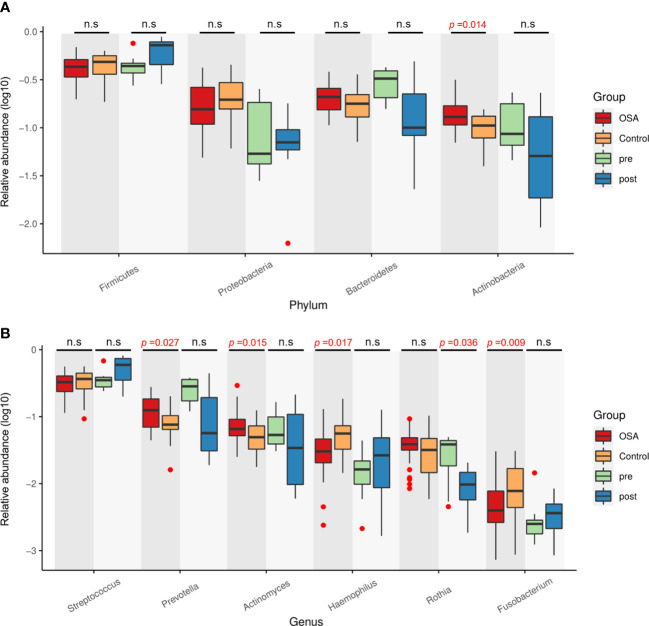
Relative abundance of four phyla and six genera showing consistent changing patterns in cross-sectional samples and longitudinal samples. Box plots showing log10-transformed relative abundances of **(A)** phylum and **(B)** genus. Boxes represent the interquartile ranges. Lines inside the boxes denote medians. Taxa are ranked in order of relative abundance from highest to lowest. *p*-Values from Mann–Whitney U-test are indicated in the figure. n.s, not significant.

Next, the relative abundances of salivary microbiota in the OSA group and control group in cross-sectional samples and the pre-treatment group and post-treatment group in longitudinal samples were compared at the phylum level and genus level. Four phyla (**Figure 8A**) and six genera (**Figure 8B**) that had consistent changing patterns in the OSA group or pre-treatment group and control group or post-treatment group were summarized and shown in box plots. The changing trend of genus *Prevotella* was found to be consistent between cross-sectional samples and longitudinal samples (**Figure 8B**). Genus *Prevotella* was significantly enriched in the OSA group (*p* = 0.027), and it was also more abundant in the pre-treatment group (*p* = 0.083).

**Figure 9:**
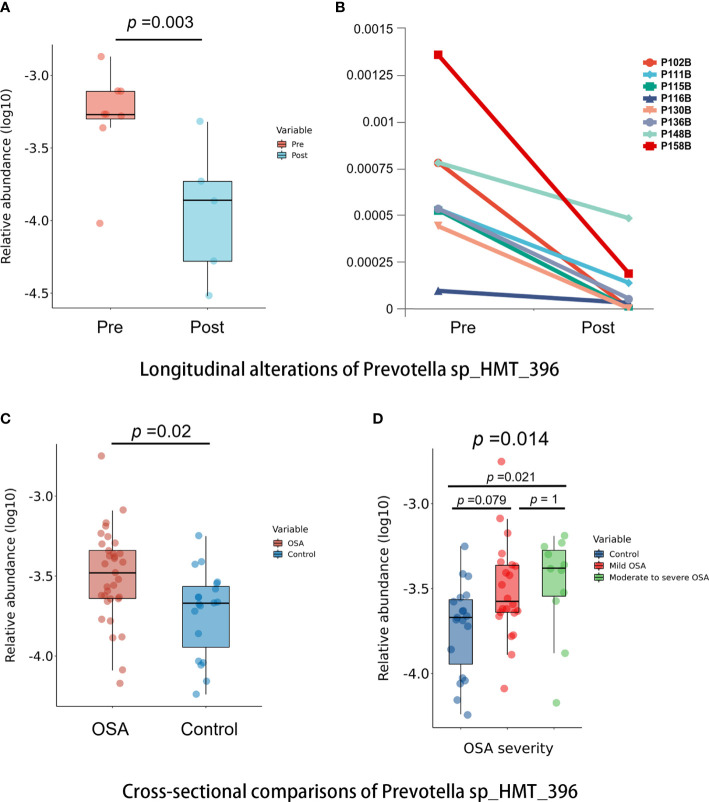
Comparisons of *Prevotella sp_HMT_396* in cross-sectional and longitudinal samples. **(A)** Box plots showing pre-treatment group had significantly higher abundance of *Prevotella sp_HMT_396* than post-treatment group in longitudinal samples (*p* = 0.003, Mann–Whitney U-test). **(B)** Line charts showing the changes of *Prevotella sp_HMT_396* of each obstructive sleep apnea (OSA) child in pre-treatment group and post-treatment group. **(C)** Box plots showing OSA group had significantly higher abundance of *Prevotella sp_HMT_396* than control group in cross-sectional samples (*p* = 0.02, Mann–Whitney U-test). **(D)** Relative abundance of *Prevotella sp_HMT_396* is significantly different among different levels of OSA severity in cross-sectional samples (*p* = 0.014, Kruskal–Wallis test).

Among all taxa, we found one species, namely, *Prevotella sp_HMT_396*, that was statistically enriched in both the OSA group and pre-treatment group (*p* < 0.05, Mann–Whitney U-test, **Figures 9A, C**). Moreover, the relative abundance of *Prevotella sp_HMT_396* increased significantly with the severity of OSA in our cross-sectional samples (*p* = 0.014, Kruskal–Wallis test, **Figure 9D**). Pairwise comparisons showed that there was a significant difference between the control group and moderate-to-severe OSA group (adjusted *p* = 0.021, **Figure 9D**). After adenotonsillectomy, the relative abundance of *Prevotella sp_HMT_396* in eight patients all decreased to varying degrees in the 1-year follow-up (**Figure 9B**).

## Discussion

Microbiota composition changes have been associated with numerous diseases (Lloyd-Price et al., 2016). In the past decades, there was increasing evidence of the association between OSA and altered microbiota composition at various locations within the human body (Cai et al., 2021), which could have important implications in understanding the pathophysiology of OSA and OSA-induced complications and in facilitating early diagnosis and treatment (Zhang et al., 2021). As a circulating fluid, saliva is painless and non-invasive to collect (Pappa et al., 2019), relatively stable over time (Belstrøm, 2020; Wang et al., 2020), and especially suitable for infants and children (Mira, 2018). Saliva microbiota is believed to serve as a potential biomarker in the future (Mira, 2018; Li et al., 2021), while limited studies have focused on the oral microbiota of OSA children (Xu et al., 2018; Davidovich et al., 2021). Davidovich et al. compared *streptococci* and *lactobacilli* counts between OSA children and controls by the method of chair-side tests, which found that *streptococci* and *lactobacilli* levels were higher in OSA children, while this method could only detect a limited number of bacteria and could not demonstrate the overall profiles of microbiota in the oral cavity (Davidovich et al., 2021). Another study by Xu et al. used buccal swabs as a representative of the oral cavity to analyze microbiota between OSA children and healthy controls with a 16S rRNA gene sequencing approach that magnificently enhanced microbiota profiling (Xu et al., 2018). The results indicated that the oral microbiota composition was significantly perturbed in OSA compared with controls. They found five genera (*Veillonella*, *Prevotella*, *Mogibacterium*, *Campylobacter*, and *Butyrivibrio*) were more abundant in OSA children.

To the best of our knowledge, this is the first report investigating microbial changes before and after OSA treatment in children. Adenotonsillectomy is recommended as a frst-line treatment for children with adenotonsillar hypertrophy (Marcus et al., 2012). However, little is known about microbial alterations before and after OSA treatment. Moreover, the quality of our samples was strictly guaranteed because all the children in the OSA group underwent PSG examination in the present study, which is the gold standard for diagnosis but has not been performed or reported in most studies related to OSA in children (Nistico et al., 2011; Dirain et al., 2017; Johnston et al., 2018; Collado et al., 2019; Galli et al., 2020; Kim and Min, 2020; Sarmiento Varón et al., 2021; Stapleton et al., 2021).

### Salivary bacterial diversity in pediatric obstructive sleep apnea

In the present study, we concluded that the salivary microbiota of pediatric OSA patients had a significantly higher α-diversity, indicating a higher richness and evenness in the salivary microbiota of OSA children. In the research conducted by Xu et al., who found that the Shannon diversity index in the microbiota of buccal swabs was higher in OSA children than in controls, they came to a similar conclusion (Xu et al., 2018). However, high α-diversity is generally considered to be a healthy state (Lloyd-Price et al., 2016; Gilbert et al., 2018). Indeed, Collado et al. (Collado et al., 2019) and Valentini et al. (Valentini et al., 2020) found that OSA children had a decreased α-diversity with respect to controls in the gut microbiota of stool samples. One possible explanation is that the environment of the oral cavity is more complex than that of the intestinal tract owing to their different sites. The oral cavity is connected directly with the outside world, making it more susceptible to environmental factors, and is therefore affected not only by diseases.

Meanwhile, our results of β-diversity revealed distinct differences in salivary microbiota between the OSA group and controls, which was consistent with Xu et al. (Xu et al., 2018). We further compared the Bray–Curtis dissimilarities (abundance-weighted) and Jaccard distances (composition-weighted) in β-diversity, and the results showed that the salivary microbiota of OSA children and controls had significant differences and could be clearly distinguished when taking bacterial composition alone into consideration. However, our longitudinal samples showed that there was a more significant change in the abundance other than composition in salivary microbiota 1 year after adenotonsillectomy, illustrating that adenotonsillectomy did not significantly alter the salivary microbiota composition. Future studies should collect samples in a time series in order to observe the alterations. Researchers are suggested to pay attention to residual mouth-breathing habits after adenotonsillectomy, which might also have an impact on the salivary microbiota.

### Characteristic salivary bacteria in pediatric obstructive sleep apnea

The presence of OSA was well reflected by the combination of some bacteria in this study. Pediatric OSA is mainly caused by adenotonsillar hypertrophy and is characterized by a chronic systemic inflammatory state (Maniaci et al., 2021), which could alter the salivary microbiota in children. In the same way, factors secondary to OSA such as habitual mouth-breathing and changes in blood oxygen saturation could also lead to microbial alteration. Cai et al. pointed out a bidirectional relationship between OSA and microbiota composition in the latest review (Cai et al., 2021), indicating that changes in microbiota might be either the cause or the effect of OSA. In the present study, 16S rRNA gene sequencing and bioinformatics analysis did not attempt to uncover a cause-and-effect relationship.

In our study, we found that genus *Prevotella* had a significantly higher abundance in the OSA group compared with controls (*p* = 0.027). This trend was verified in the longitudinal samples in which the relative abundance of *Prevotella* has a trend of decline 1 year after adenotonsillectomy although without a statistical difference (*p* = 0.083). Moreover, *Prevotella sp_HMT_396* was found to be significantly enriched in both the OSA group (*p* = 0.02) and pre-treatment group (*p* = 0.003) and less abundance in the control group and post-treatment group. At the same time, we found that *Prevotella sp_HMT_396* might be related to the severity of OSA, because it had significantly higher levels, as OSA severity increased and its abundance decreased 1 year after adenotonsillectomy in each OSA child. In previous studies, Dirain et al. revealed that *Prevotella* was significantly more common on adenoids in children with OSA than in those with recurrent acute otitis media (AOM) by 454 pyrosequencing analysis (Dirain et al., 2017). *Prevotella* has been recognized as one of the core anaerobic genera in the oral microbiome (Brook et al., 2000). In fact, *Prevotella* has been associated with localized and systemic inflammatory diseases. For example, the increased abundance of *Prevotella* has been linked with periodontitis, augmented T helper type 17 (Th17) ‐mediated mucosal inflammation, rheumatoid arthritis, inflammatory autoimmune diseases, metabolic disorders, and low-grade systemic inflammation (Larsen, 2017; Tett et al., 2021), suggesting the inflammatory properties of *Prevotella*. OSA is associated with the occurrence of intermittent hypoxia and fragmented sleep, which could lead to oxidative stress and low-grade inflammatory responses (Tan et al., 2013; Cai et al., 2021). In the current study, the higher abundance of *Prevotella* in OSA children could be related to inflammation as well, although direct causation is uncertain, which calls for further studies on *Prevotella* to clarify its impact on pediatric OSA.

*Actinomyces* had a significantly higher abundance in the OSA group compared with controls (*p* = 0.015). The relative abundance of *Actinomyces* tended to decrease after adenotonsillectomy but without statistical significance, as demonstrated in **Figure 8**, which might be due to the small number of cases followed up. Previous studies demonstrated a strong association between tonsillar hypertrophy and the presence of *Actinomyces* by hematoxylin and eosin staining, indicating the potential etiologic role of *Actinomyces* in the development of obstructive tonsillar hypertrophy (Bhargava et al., 2001; Ozgursoy et al., 2008; Riffat and Walker, 2009; Kutluhan et al., 2011). Similarly, Riffat et al. performed a histological analysis of 1,213 tonsillectomy specimens from children aged less than 16 years and noted a statistically significant higher rate of *Actinomyces* colonization in children with sleep-disordered breathing (SDB) compared to recurrent tonsillitis (Riffat and Walker, 2009). Other researchers have come to similar conclusions (Bhargava et al., 2001; Ozgursoy et al., 2008; Kutluhan et al., 2011).

In the present study, *Lactobacillus* ranked first in importance as shown in random forest analysis, indicating that *Lactobacillus* was the most important indicator to distinguish OSA from controls. Moreover, *Lactobacillus* had a significantly higher abundance in the OSA group compared with controls (*p* < 0.001). A recent study by Davidovich et al. compared oral features of children with OSA and healthy controls (Davidovich et al., 2021), and they found that OSA children had significantly lower oral pH values than controls (*p* < 0.0001). A low pH favors the enrichment of acid-producing and acid-tolerant bacteria in the oral cavity such as species of the genera *Streptococcus*, *Actinomyces*, *Lactobacillus*, and *Bifidobacterium* (Aas et al., 2008; Richards et al., 2017; Boisen et al., 2021). Meanwhile, a significantly high level of salivary *lactobacilli* counts in OSA children in the study by Davidovich et al. concurs with the present study. Interestingly, one study by Sharma et al. also concurs with our findings, which showed that the salivary microbiota of patients with primary Sjögren’s syndrome (characterized by dry mouth) was enriched in *Lactobacillus*, *Bifidobacterium*, and *Dialister* when compared with controls (Sharma et al., 2020). Thus, the enrichment of *Lactobacillus* in the saliva of OSA children might be related to small changes in pH and dry mouth caused by mouth-breathing. It is worth mentioning that the effects of poor oral health status might be a confounding factor (Su et al., 2011; Rossi et al., 2019). In our study, we have excluded the difference in oral health status between the OSA group and control group by conducting oral examinations on each subject in the present study.

Not coincidentally, genus *Escherichia* and species *E. coli* were substantially abundant in the OSA group compared with controls (*p* < 0.001), and *Escherichia* was identified as the second most important genus to classify OSA patients from the controls according to the result of random forest. This might also have an association with mouth-breathing because studies have shown that the incidence of dry mouth upon awakening is much higher in OSA patients versus primary snorers (Oksenberg et al., 2006). Meanwhile, the colonization of *E. coli* was found to be an important change in the oral microbiota of people with dry mouths (Wade, 2021).

In this study, the OSA group showed a significantly higher abundance of genus Bifidobacterium (p < 0.001), species B. longum (p < 0.001), and species B. breve (p = 0.003) than control group. Bifidobacterium usually belongs to probiotic genera, just like *Lactobacillus*. Even though *Lactobacillus* and *Bifidobacterium* could play a beneficial role (Alanzi et al., 2018; Hasslöf and Stecksén-Blicks, 2020), not all of their species provide beneficial effects (Bizzini et al., 2012). The relative abundance of *Lactobacillus* and *Bifidobacterium* was found to increase in gut microbiota in OSA-induced hypertension models (Zhang et al., 2021), and they have also been recognized as novel caries-associated bacteria (Beighton et al., 2008; Manome et al., 2019).

### Limitations

There were several limitations in the present study. First, this study could provide information only on comparisons between groups, in which the causality could not be determined. Microbiota composition changes in pediatric OSA patients might be a cause, a consequence, or both. This study was unable to establish a causal relationship between OSA and salivary microbiota. Second, our sample size was small because the enrollment criteria for OSA patients were stringent and the fact that OSA children’s subjective symptoms improved significantly after treatment, which increased the difficulty of follow-up. Third, saliva flow rate and saliva pH have not been measured in the present study, which needs to be paid attention to in future studies. Fourth, subjects in the control group were selected through PSQ-SRBD, the answers to which might be subject to recall bias. Although PSQ-SRBD is well validated as a predictor of OSA (Li et al., 2016; Li et al., 2018), our control group did not undergo PSG in consideration of the cost, medical resource constraints, and ethical concerns. We mitigated this by enrolling participants with scores less than 3 (threshold = 7) to capture those at the lowest reported risk.

## Conclusion

In OSA children, a higher microbial diversity as well as an enrichment of OSA characteristic genera (*Prevotella*, *Actinomyces*, *Lactobacillus*, *Escherichia*, and *Bifidobacterium*) were identified in the saliva of OSA children, showing the potential to serve as OSA salivary biomarkers. Among these characteristic taxa, the relationship between *Prevotella* spp. and OSA is worthy of future studies.

## Data availability statement

The data presented in the study are deposited in the Sequence Read Archive (https://www.ncbi.nlm.nih.gov/), accession number PRJNA836180.

## Ethics statement

This study was reviewed and approved by Institutional Review Board of Peking University Hospital of Stomatology. Written informed consent to participate in this study was provided by the participants’ legal guardian/next of kin.

## Author contributions

XH, ZX, and XMG contributed to the conception and design of the study. XH and XC contributed to sample collections, DNA extraction, and bioinformatics and statistical analyses. XG and YX performed the acquisition of data. XH wrote the first draft of the manuscript, and XG and YX prepared the tables and figures. XMG, ZX, XG, XC, and YX took part in revising and reviewing the manuscript. All authors contributed to manuscript revision and read and approved the submitted version.

## Funding

This study was funded by the National Natural Science Foundation of China (82170102) and National Program for Multidisciplinary Cooperative Treatment on Major Diseases (PKUSSNMP-201902).

## Acknowledgments

We thank all the children and their families who participated in this study during the coronavirus disease 2019 (COVID-19) pandemic, during which the difficulty of follow-up has greatly increased. We gratefully acknowledge Li Zheng from the Sleep Center of Beijing Children’s Hospital for the sampling assistance. We thank Chi Zhang from Sleep Medicine Center in Peking University People’s Hospital for technical support on sample size estimation.

## Conflict of interest

All authors declare that the research was conducted in the absence of any commercial or financial relationships that could be construed as a potential conflict of interest.

## Publisher’s note

All claims expressed in this article are solely those of the authors and do not necessarily represent those of their affiliated organizations, or those of the publisher, the editors and the reviewers. Any product that may be evaluated in this article, or claim that may be made by its manufacturer, is not guaranteed or endorsed by the publisher.
