# Supplementary material for: Characteristics of salivary microbiota in children with obstructive sleep apnea: A prospective study with polysomnography
Source: Front Cell Infect Microbiol. 2022 Aug 29;12:945284. doi: 10.3389/fcimb.2022.945284 (PMC9465092; doi:10.3389/fcimb.2022.945284)
Supplement: Supplementary file 1 [file Table_1.docx]

Supplementary Material

**Supplementary Table 1.** Effect sizes (ω^2^) for PERMANOVA power of 80% and 90% with varied sample sizes.

| Sample numbers in each group | ω^2^ / P-value | |
| --- | --- | --- |
|  | power 80% | power 90% |
| 5 | 0.0766 | 0.092 |
| 10 | 0.0311 | 0.0411 |
| 20 | 0.0202 | 0.0269 |
| 30 | 0.00907 | 0.013 |
| 40 | 0.00645 | 0.0105 |

Human Microbiome Project (HMP) dataset was used for distance matrix simulation. “Micropower” package (http://github.com/brendankelly/micropower) was used to assess the effect size and statistical power.
